# The episodic random utility model unifies time trade-off and discrete choice approaches in health state valuation

**DOI:** 10.1186/1478-7954-7-3

**Published:** 2009-01-13

**Authors:** Benjamin M Craig, Jan JV Busschbach

**Affiliations:** 1 Health Outcomes & Behavior Program, Moffitt Cancer Center, 12902 Magnolia Drive, MRC-CANCONT, Tampa, Florida 33612-9416, USA; 2 Department of Economics, University of South Florida, Fowler St, Tampa, Florida, USA; 3 Department of Medical Psychology and Psychotherapy, Erasmus MC, Rotterdam, The Netherlands

## Abstract

**Background:**

To present an episodic random utility model that unifies time trade-off and discrete choice approaches in health state valuation.

**Methods:**

First, we introduce two alternative random utility models (RUMs) for health preferences: the episodic RUM and the more common instant RUM. For the interpretation of time trade-off (TTO) responses, we show that the episodic model implies a coefficient estimator, and the instant model implies a mean slope estimator. Secondly, we demonstrate these estimators and the differences between the estimates for 42 health states using TTO responses from the seminal Measurement and Valuation in Health (MVH) study conducted in the United Kingdom. Mean slopes are estimates with and without Dolan's transformation of worse-than-death (WTD) responses. Finally, we demonstrate an exploded probit estimator, an extension of the coefficient estimator for discrete choice data that accommodates both TTO and rank responses.

**Results:**

By construction, mean slopes are less than or equal to coefficients, because slopes are fractions and, therefore, magnify downward errors in WTD responses. The Dolan transformation of WTD responses causes mean slopes to increase in similarity to coefficient estimates, yet they are not equivalent (i.e., absolute mean difference = 0.179). Unlike mean slopes, coefficient estimates demonstrate strong concordance with rank-based predictions (Lin's rho = 0.91). Combining TTO and rank responses under the exploded probit model improves the identification of health state values, decreasing the average width of confidence intervals from 0.057 to 0.041 compared to TTO only results.

**Conclusion:**

The episodic RUM expands upon the theoretical framework underlying health state valuation and contributes to health econometrics by motivating the selection of coefficient and exploded probit estimators for the analysis of TTO and rank responses. In future MVH surveys, sample size requirements may be reduced through the incorporation of multiple responses under a single estimator.

## Background

Health state valuation studies using the time trade-off (TTO) approach lack a sound theoretical framework for the incorporation of worse than death (WTD) responses. Furthermore, TTO responses may be considered a form of discrete choice (i.e., expressions of a tie between two alternative scenarios); yet, no valuation study has applied discrete choice estimators to TTO data. In this paper, we introduce an episodic random utility model (RUM) and two novel estimators for health state valuation. We show that the assumption of the episodic RUM theoretically and econometrically unifies TTO and other discrete choice approaches.

**Figure 1:**
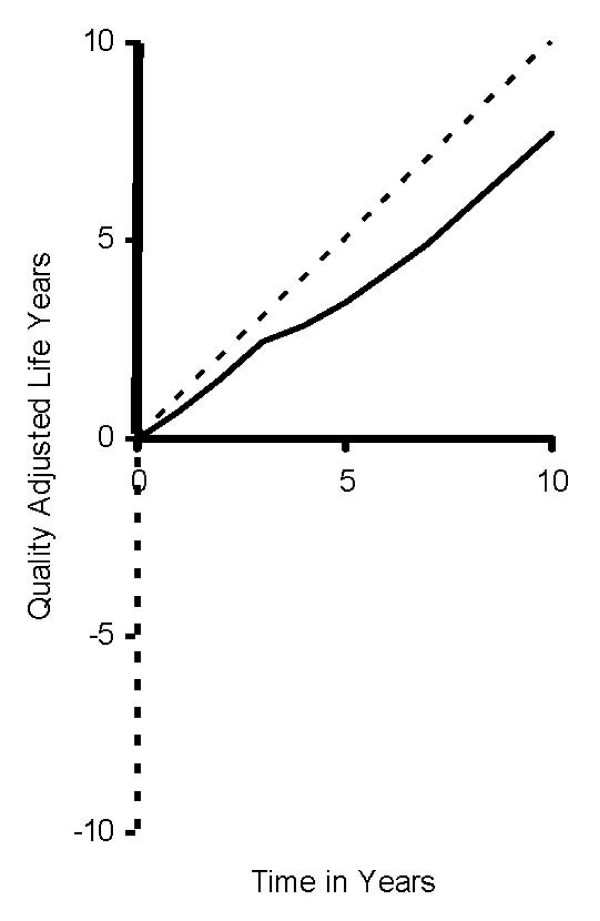
**QALY Space: Accumulation of Quality-Adjusted Life Years over Time**.

Estimating the value assigned to an episode of health is the main purpose of the EuroQol Group, and the focus of this paper. In Figure 1, the solid line represents the accumulation of quality adjusted life years (QALYs) for a person over time. The slope of the line represents the instantaneous value of health at each point in time. For example, the kink in the line suggests that at around the third year, the person's health was poor, as represented by a shallow slope; however, her health improved, and after ten years, she accumulated around seven QALYs. In other words, the value assigned to her decade-long health episode is seven.

As described by Torrance in 1982, values of better than dead (BTD) states are bounded by the values of optimal health (1.00) and dead (0). WTD states may be as large as minus infinity [1]. In Figure 1, a person's "spaghetti" line may lie anywhere between the dotted lines, but the slope of the spaghetti line must remain between one and minus infinity. The potential for an infinitely negative slope poses a fundamental challenge in the estimation of QALYs using TTO, standard gamble (SG), person trade-off (PTO), or any other discrete-choice approach. In TTO, the conventional approach to QALY estimation entails an average of positive and negative slopes (i.e., mean slope estimator); a similar process is applied in SG and PTO. An often noted problem is that the influence of negative slopes can be so massive (e.g., -39 in the MVH study) that the mean slopes appear much too low, well outside the reasonable range of face validity within the QALY concept.

Confronted with this threat to face validity, researchers typically manipulate WTD response data, arbitrarily increasing the negative slopes and imposing an ad-hoc boundary of negative one on the slopes. The boundary of negative one reduces the influence of negative slopes on the mean slope and gives an appealing mirror image for the valuations space above zero. Nevertheless, critics from early on have warned that there is no theoretical justification for the value of negative one, which means the truncated scale may not represent 'utility' [2]. Changing data to improve face validity is generally frowned upon, even in the case of outliers.

A similar health econometrics discussion has taken place on cost analyses, revealing that the transformation of positive outliers has a large effect on the mean cost per patient. At the 2008 American Society of Health Economists, John Mullahy compared the role of a health econometrician to that of an anatomist, dissecting data in an Aristotelian fashion [3]. In his lecture, "Anatomy of Healthcare Cost Distributions," he dismantled the thick upper tail of a common cost distribution and discussed its possible interpretations. Likewise, health state valuation studies continuously re-examine the theoretical framework that guides estimator selection and the best approach to address results with poor face validity.

In pursuit of a justification for this ad hoc transformation, studies report that respondents find it more difficult and make more errors estimating negative values than estimating positive values, especially in TTO tasks [4]. These psychometric complications are reflected in the high variance of negative values, the low discriminating power of negative values, and the discontinued scale around the value of death, otherwise known as the 'gap-effect' [5-7]. While the evidence on the influence of state-specific heteroskedasticity is mounting, there is not yet a clear and coherent framework for combining BTD and WTD TTO responses.

Recently, there has been considerable interest in estimating health state values from ranking exercises suitable for QALY calculations [4,5,8,9]. Ranking is seen as a relatively easy valuation method, like the visual analogue scale (VAS), and shown to render predictions that are concordant with (if not identical to) VAS predictions [5]. The advantage of ranking versus VAS is a well developed theoretical foundation in Item Response Theory without the response spreading and context effects associated with VAS [10]. Unlike VAS, ranking is a choice-based approach, which provides a basis for its merger with economic oriented choice-based methods, like TTO and SG. A drawback for both ranking and VAS is their unclear relation to health state values on the QALY scale, a relation which is better described for TTO and SG.

A theoretically driven model that reduces the difference between a psychometrically strong method (e.g., ranking) and a method with a strong link to utility theory (e.g., TTO) has the potential to revolutionize the field of health state valuation. This model would increase the 'convergent validity' of related psychometric and econometric methods, and therefore, enhance the 'construct validity' of these methods [11]. In the absence of a 'gold standard' in health state valuation, such an increase in convergent validity would advance our understanding regarding the latent construct of quality of life and its assessment. Furthermore, if a model reduces dependence on arbitrary deviations from utility theory, such as negating the use of ad hoc corrections of WTD responses in the QALY paradigm, the model would promote face validity. Lastly, such a model might further improve upon the validity of QALYs by integrating the benefits of psychometric and econometric methods under a single statistical estimator.

In this paper, we introduce an episodic random utility model (RUM) as such a theoretical framework. This model not only allows for the comparisons between rank and TTO predictions within a common estimator, it resolves key econometric and psychometric issues that inhibit TTO-based valuation. In introducing this model, the difficulties with the face validity of WTD responses are addressed in a way that is theoretically coherent for the fields of economics and psychometrics, and improves upon the convergent validity between TTO and rank-based predictions. For purposes of illustration, the conventional and episodic RUMs are estimated using the Measurement and Valuation of Health (MVH) study data from the United Kingdom (UK) [12-14].

## Methods

### Episodic and Instant Random Utility Models (RUMs)

The utility of a health state, *j*, over time, *t*, for an individual, *i*, is random and may be represented by either:

(1) $$U_{ij}(t)=\{\begin{array}{ll} \mu_{j}t+\varepsilon_{ij} & Episodic\;RUM\\ \left(\mu_{j}+\varepsilon_{ij}\right)t=\mu_{j}t+\varepsilon_{ij}t & Instant\;RUM \end{array}$$

In the episodic RUM, the error, *ε*_*ij*_, represents variability in the value of an episode. For example, Figure 1 has time on the x-axis and utility on the y-axis, so the error would be distributed vertically along the y-axis. The second model is an instant RUM, which suggests a random slope. Its error, *ε*_*ij*_, represents variability in the value of an instantaneous state, not the episode. The instant RUM is the theoretical basis underlying the mean slope estimator, the conventional approach to health state valuation studies.

The instant RUM would be equivalent to an episodic RUM if we were to assume that the magnitude of error is proportional to the duration of the episode. In other words, more time in state *j *coincides with more error in the valuation. However, each model assumes that errors have equal variances. This difference is subtle, but highly influential in cases where there are WTD TTO responses. In WTD responses, the respondent's choice of time in optimal health changes the amount of time in state *j*, thus, changing the amount of error under the instant RUM. For example, if the respondent equates the state to "immediate death" (t = 0), according to the instant RUM model, there is no error in this response.

Both models assume that the utility of dead for any duration is zero (i.e., U_dead_(t) = 0), and the utility of optimal health for any duration equals the duration (i.e., U_optimal_(t) = t) [5]. Both models assume constant proportionality: the expected utility of a health state is proportional to its duration, *t*, and the expected error is zero. State-specific components and errors may depend on the duration (e.g., *μ*_*j*_(*t*)) [15,16]; however, questions concerning duration effects in health state valuation are outside the scope of this paper and left to be examined in future work.

### Interpretations of TTO responses

As part of the TTO task in the MVH study, respondents provide either a BTD or WTD response for each hypothetical health state, *j*; however, the interpretation of the responses depends on the RUM. If ten years in a health state, *j*, is better than "immediate death," the respondent determines the duration in optimal health, *t*_1_, such that:

(2) $$U_{ij}(10)=U_{ioptimal}(t_{1})\Rightarrow \{\begin{array}{ll} t_{1}=\mu_{j}10+\varepsilon_{ij} & Episodic\;RUM\\ t_{1}/10=\mu_{j}+\varepsilon_{ij} & Instant\;RUM \end{array}$$

The interpretation of the BTD response, *t*_1_, is for all intensive purposes equivalent under episodic and instant RUMs, because the amount of time in state *j *is equal regardless of response (i.e., ten years).

On the other hand, if ten years in the health state, *j*, is worse than dead (WTD), the respondent determines the duration in optimal health, *t*_2_, such that:

(3) $$U_{ij}(10-t_{2})+U_{ioptimal}(t_{2})=U_{idead}(10)\Rightarrow \{\begin{array}{ll} -t_{2}=\mu_{j}(10-t_{2})+\varepsilon_{ij} & Episodic\;RUM\\ -t_{2}/(10-t_{2})=\mu_{j}+\varepsilon_{ij} & Instant\;RUM \end{array}$$

The interpretation of the WTD response, *t*_2_, differs greatly between the episodic and instant RUM estimates.

### RUM Estimators

The purpose of a RUM estimator is to find the estimate for the state-specific component, *μ*_*j*_, that best fits the sample of responses (i.e. minimizes the error). RUMs do not imply a specific error distribution. Errors have expectation zero, are uncorrelated, and have equal variances. Under these three assumptions, the Gauss Markov theorem states that the best (i.e., minimum variance) estimator of the state-specific component, *μ*_*j*_, is a mean slope under the instant RUM approach and a coefficient under the episodic RUM [17].

(4) $${\hat{\mu }}_{j}=\{\begin{array}{ll} \left(\sum_{BTD}10t_{1i}+\sum_{WTD}-t_{2i}\left(10-t_{2i}\right)\right)/\left(\sum_{BTD}10^{2}+\sum_{WTD}{\left(10-t_{2i}\right)}^{2}\right) & Episodic\;RUM\\ \frac{1}{N}\left(\sum_{BTD}t_{1i}/10+\sum_{WTD}-t_{2i}/\left(10-t_{2i}\right)\right) & Instant\;RUM \end{array}$$

Both estimators are non-parametric, and they are equivalent if the sample only includes BTD responses, *t*_1_. The instant RUM estimator is a mean slope (Figure 1), and because slopes can be exceptionally negative (e.g., -39), the mean slope estimator is not robust to small changes in the error term. The episodic RUM estimator is a fraction of weighted sums, creating additional stability.

Beginning in the mid 1990's, the field of economic evaluations faced a similar choice between estimators [18,19]. The emergence of patient-level data led to the question of whether to use the mean ratio (i.e., mean slope) or the mean cost over the mean effectiveness as the estimator of the incremental cost-effectiveness ratio (ICER). Like in our case, if incremental effectiveness approaches zero for any patient, the patient's ICER blows up together with the mean. As such, ratio statistics are not widely used in cost-effectiveness research.

A parallel argument in favor of the coefficient estimator comes from psychometrics. The coefficient estimator is motivated by economic theory (i.e., episodic RUM). However, measurement theory also implies the same estimator with a slightly different interpretation: when respondents provide the amount of time in optimal health, they may respond with some error (t + *ε*) [19]. The coefficient estimator accommodates such response error.

Nevertheless, the mean slope estimator is the conventional approach to health state valuation studies using discrete choice methods (i.e., TTO, SG, PTO, etc.). In an effort to improve the face validity of instant RUM predictions, Dolan replaced the negative slopes with -t_2_/10, while Shaw and colleagues divided the negative slopes by a constant (i.e., 39) [12,20]. Each transformation attenuates the magnifying effects in the slopes by bounding them to be greater than negative one. In the economic evaluation analogy, Dolan's transformation is like changing the incremental effectiveness to the maximum, 10 years, when the patient's ICER is negative. By construction, the Dolan approach will produce estimates greater than the unadjusted mean slope, but less than the coefficient if there are any WTD responses (mathematical proof available upon request). These arbitrary manipulations are not nested within either the instant or episodic RUMs, or within any other utility or psychometric theory [2].

### Mixing TTO, Rank, and RUM

While TTO estimation does not require further specification to produce consistent results, it may be more efficient to assume the errors are normally distributed. This assumption allows for maximum likelihood estimation and, more importantly, the merger of rank and TTO responses under a single estimator.

Craig, Busschbach, and Salomon demonstrated that ranks can be decomposed into a series of pair-wise comparisons for rank-based health state valuation using an exploded probit model [5,17]. Because hypothetical states continue for ten years (i.e., *t *does not vary) in all EQ-5D rank responses within the UK MVH-protocol, their model estimates agree with either the episodic or instant RUMs. Under the assumption of normally distributed errors, the probability of dominance for each pair-wise comparison in the rank responses is represented by:

(5) $$\mathrm{Pr}\left(U_{ij}\left(t_{j}\right)>U_{ik}\left(t_{k}\right)\right)=\mathrm{Pr}\left(\varepsilon_{ij}-\varepsilon_{ik}<\mu_{j}t_{j}-\mu_{k}t_{k}\right)=\Phi \left(\frac{\mu_{j}t_{j}-\mu_{k}t_{k}}{\sqrt{\sigma_{j}^{2}+\sigma_{k}^{2}}}\right)$$

The exploded probit estimation can predict the state-specific components and variances. While health states clearly have different expected utilities, differences in variances (i.e., *σ*_j _≠ *σ*_k_) have little effect on the predicted values as demonstrated by Craig, Busschbach, and Salomon [5]. Therefore, in this article, we estimated a homoskedastic probit model using rank responses and predicted values for 42 health states on the QALY scale with fixed anchors for comparison. TTO responses were used to predict the OLS episodic RUM.

The rank-based estimator (equation 5) required slight modification (equation 6) to incorporate both the episodic RUM and TTO responses. In rank responses, ties occur when a respondent considers two or more states to be equivalent. TTO responses can also be described as equivalences between two hypothetical scenarios (equations 2 and 3). Therefore, TTO responses can be incorporated into the same exploded probit model using Efron's method for ties in rank responses[21]. Specifically, the probability of a TTO response is:

(6) $$\mathrm{Pr}\left(\varepsilon_{ij}=\mu_{j}x_{j}-y_{j}\right)\Phi {\left(\frac{y_{j}-\mu_{j}x_{j}}{\sqrt{\sigma_{j}^{2}}}\right)}^{0.5}\Phi {\left(\frac{\mu_{j}x_{j}-y_{j}}{\sqrt{\sigma_{j}^{2}}}\right)}^{0.5}$$

where y equals t_1 _if BTD, or -*t*_2 _if WTD, and x equals 10 if BTD, or (10-*t*_2_) if WTD. This is equivalent to a simple linear regression with no constant and an assumption of normally distributed errors with state-specific variances. A central advantage of the exploded probit is the estimator can accommodate both TTO and rank responses.

Caution is warranted when merging responses from different valuation techniques into a single estimator. While the estimation of state-specific components, *μ*_j_, may benefit greatly from the added information, it remains unclear whether the TTO variance is equal to the variance found in rank responses. Completion of the TTO task entails a greater cognitive burden for respondents, which may result in greater errors. In the combined estimator, a separate variance parameter describing the difference between the method-specific variances is included for rank responses.

In combining TTO and rank responses within a single estimation, we increase the power of valuation studies that explore preferences of respondents using both TTO and rank responses. In most valuation studies done on the basis of the MVH protocol, both TTO and rank were administrated. A problem might be that there are more ranked pairs than TTO responses. To impose balance across methods, we assigned the pair-wise comparisons a reduced weight equal to the respondent's number of hypothesized non-anchor states over the respondent's number of pair-wise comparisons. As a result, each respondent's set of decomposed rank responses received the same weight in the maximum likelihood estimation as their set of TTO responses. The estimator accounts for both sources of information equitably.

## Results

### United Kingdom Measurement and Valuation of Health (MVH) Study

In 1993, the University of York administered 3395 interviews with a response rate of 64%, and collected values of 42 EQ-5D health states and the state of unconsciousness [12-14]. The MVH protocol, developed for the aforementioned study, describes a face-to-face interview that can be separated into several sections. First, the respondents are asked to describe their own health using the EQ-5D descriptive system. Then, the respondents rank 15 cards each describing a health state. This set of 15 health state cards always includes the anchor states, optimal health (11111) and immediate death. The respondents are instructed to assume that the duration of the health state is 10 years and followed by death. After the ranking exercise, the subjects are asked to place each card on the EQ-VAS, often referred to as the EuroQol "thermometer." After the EQ-VAS valuation section, the deck of health state cards is reshuffled, and 13 health states are valued using the TTO method. The two missing states are 11111 and 'immediate death' as these states cannot be valued directly using the standard TTO, because they anchor the TTO scale. The TTO-interview is complemented by a visual aid, specifically a TTO-probe board that graphically displays the difference in life years between health states. As previously described, the TTO task produces either *t*_1 _or *t*_2 _responses, each of which describes a compensating amount of time in the optimal health state.

For the TTO and rank analytical sample (N = 3,333 and 3,355, respectively), respondents were excluded for a particular method (1) if only one or two states were valued (other than 11111, "immediate death," and "unconscious"); (2) if all states were given the same value; or (3) if all states were valued worse than "immediate death." In addition, respondents were excluded from the rank sample if they ranked death equivalent to optimal health. These four criteria motivated the exclusion of 1.8% of the rank respondents and 1.2% of the TTO respondents.

### Comparison between Instant and Episodic RUM

**Figure 2:**
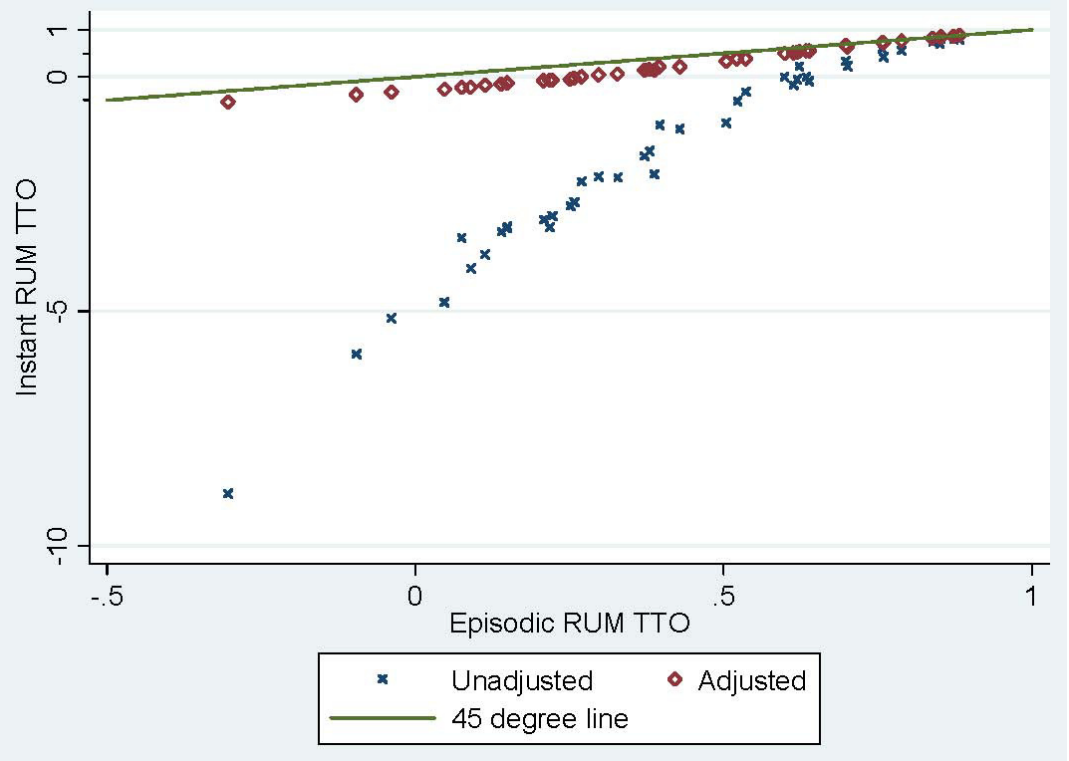
Comparison of Instant and Episodic RUM TTO Estimates for 42 EQ-5D Health States with and without the Negative One Ad Hoc Boundary Adjustments * Adjustment of the TTO responses is based on Dolan's transformation of negative responses. The episodic RUM has a lowest value of just above -0.50, while the negative values of the instant RUM can be as low as -9.00.

Figure 2 illustrates the relationship between the episodic and instant RUM predictions using TTO responses for the 42 EQ-5D hypothetical health states included in the UK MVH study. As described in equation 4, the instant RUM estimates are mean slopes. These means are presented with and without Dolan's transformation of WTD responses to a bound of -1.00. The unadjusted means are substantially less than the episodic RUM predictions, depending on the quantity of WTD responses. This pattern intuitively illustrates the effect of summing fractions, instead of taking a coefficient (i.e., a fraction of sums).

Figure 2 further illustrates that the adjusted mean slopes based on Dolan's transformation of negative responses are linearly related to the coefficient estimates derived from the episodic RUM. Based on Table 1, episodic and instant predictions are correlated above 97% for Pearson's or Spearman's rho. However, unadjusted mean slopes poorly agree with coefficient estimates (Lin's rho = 0.135). On the other hand, the adjusted mean slopes moderately agree (Lin's rho = 0.841). The predictions rendered through the arbitrary correction of WTD responses are still substantially different from the episodic RUM predictions (average absolute difference = 0.179). While we recognize that the Dolan transformation improves concordance between the instant and episodic predictions, improved face validity is not a sufficient motivation for data manipulation.

**Table 1:** Correlation and Agreement between Predicted Values for 42 EQ-5D States

	Comparison between Episodic RUM Estimates using TTO responses and...			
	Instant RUM Estimates using...		Episodic RUM Estimates using...	
	Unadjusted TTO Responses	Adjusted TTO Responses*	Rank Responses	TTO ank Responses
Correlation				
Pearson's rho	0.971	0.994	0.977	0.981
Spearman's rho	0.993	0.999	0.985	0.986
				
Agreement				
Lin's rho	0.135	0.841	0.910	0.949
Mean absolute difference	2.097	0.179	0.098	0.074

* Adjustment of these TTO responses is based on Dolan's transformation of negative responses.

### Episodic RUMs using TTO, Rankings, and Both Responses

Table 1 further describes the relationship between the predictions from the three episodic RUM estimations. Coefficient estimates based on TTO responses show stronger agreement with rank-based predictions (Lin's rho = 0.910) than adjusted mean slopes (Lin's rho = 0.794). This suggests that rank responses provide similar information to TTO responses based on the episodic RUM compared to the instant RUM with Dolan's transformation of WTD responses. Convergence validity between the two methods is improved more by a theoretical coherent model, than by an ad hoc boundary of -1.00. This, in turn, increases the construct validity of both the ranking and TTO estimates for health state valuation.

**Figure 3:**
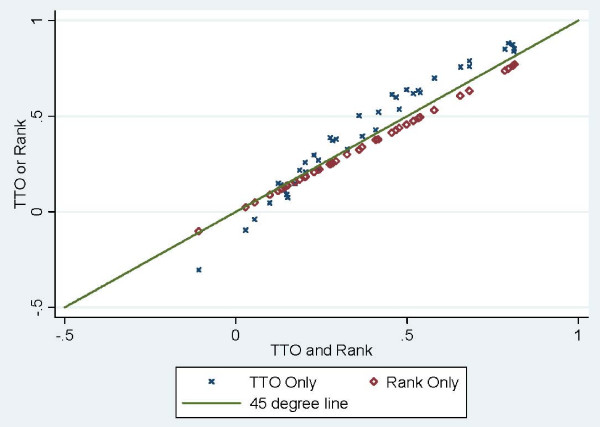
**Comparison of Episodic RUM Estimates using Single and Both Responses for 42 EQ-5D Health States**.

Figure 3 illustrates the relationship between episodic predictions. Compared to the rank-based predictions, coefficient estimates are slightly higher for mild health states and lower for states near death, which suggests the potential for duration dependence in health state valuation [15,16]. Future analysis may parameterize the duration effect and estimate the extent of adaptation. Overall, the pattern between episodic predictions suggests strong concordance.

Lastly, we compared the 95% confidence intervals between episodic RUM predictions using TTO responses, and intervals using TTO and rank responses (See Table 2). Among the 42 states, TTO confidence intervals of three states have their dual response counterparts nested within and 12 states have intervals that overlap. This discordance suggests some systematic differences between the TTO and rank-based values. In terms of interval width, the average width of the TTO interval is 0.057 with a range of 0.021 to 0.094. The average width of the dual response interval is 0.041 with a range of 0.027 to 0.047. For eleven mild health states, the TTO based interval is narrower than the dual response intervals; but for the remaining more severe states the dual response interval is narrower. The interval widths of the TTO and rank responses suggest that the use of both responses decreases the standard error in health state value predictions by around two thirds and allows for greater variability in the value of mild states.

**Table 2:** State-specific Component Estimates (*μ*_j_) by EQ-5D State, Model and Estimator

	Instant RUM						Episodic RUM								
EQ-5D State	Unadjusted TTO			Adjusted TTO			TTO			Rank			TTO and Rank		
	*μ*_j_	95% CI		*μ*_j_	95% CI		*μ*_j_	95% CI		*μ*_j_	95% CI		*μ*_j_	95% CI	
21111	0.795	0.705	0.885	0.873	0.860	0.886	0.883	0.872	0.893	0.748	0.739	0.756	0.796	0.776	0.817
11211	0.801	0.717	0.885	0.865	0.852	0.878	0.873	0.863	0.884	0.763	0.754	0.771	0.808	0.788	0.829
11121	0.809	0.746	0.873	0.847	0.834	0.861	0.853	0.840	0.865	0.770	0.762	0.779	0.814	0.793	0.834
12111	0.699	0.588	0.811	0.831	0.815	0.847	0.851	0.839	0.864	0.739	0.730	0.747	0.786	0.765	0.806
11112	0.739	0.649	0.829	0.824	0.808	0.839	0.839	0.827	0.852	0.768	0.759	0.776	0.811	0.791	0.832
12211	0.554	0.364	0.745	0.762	0.740	0.785	0.789	0.771	0.806	0.632	0.623	0.642	0.682	0.659	0.706
11122	0.421	0.200	0.642	0.719	0.694	0.745	0.760	0.742	0.778	0.633	0.624	0.642	0.682	0.658	0.705
12121	0.585	0.420	0.751	0.739	0.718	0.761	0.758	0.739	0.776	0.607	0.598	0.616	0.656	0.632	0.679
22121	0.227	-0.017	0.471	0.640	0.612	0.669	0.701	0.681	0.721	0.532	0.524	0.541	0.579	0.557	0.602
22112	0.333	0.102	0.564	0.660	0.634	0.685	0.698	0.679	0.718	0.533	0.524	0.541	0.580	0.558	0.603
11312	-0.097	-0.418	0.224	0.553	0.521	0.585	0.639	0.617	0.662	0.455	0.447	0.463	0.499	0.477	0.520
21222	-0.011	-0.303	0.281	0.546	0.515	0.578	0.634	0.613	0.655	0.489	0.481	0.498	0.533	0.510	0.555
12222	0.214	0.011	0.416	0.543	0.511	0.575	0.623	0.600	0.646	0.495	0.487	0.504	0.538	0.516	0.560
22122	-0.056	-0.353	0.241	0.530	0.497	0.562	0.620	0.597	0.642	0.475	0.467	0.484	0.518	0.496	0.540
21312	-0.173	-0.492	0.145	0.515	0.481	0.549	0.613	0.590	0.637	0.415	0.407	0.423	0.455	0.434	0.477
22222	-0.003	-0.273	0.266	0.501	0.468	0.533	0.599	0.577	0.622	0.427	0.419	0.435	0.468	0.447	0.489
11113	-0.319	-0.607	-0.030	0.389	0.351	0.427	0.536	0.509	0.563	0.440	0.432	0.448	0.477	0.456	0.498
13212	-0.517	-0.869	-0.165	0.377	0.340	0.413	0.522	0.497	0.547	0.379	0.372	0.387	0.416	0.396	0.437
13311	-0.987	-1.418	-0.556	0.329	0.290	0.368	0.504	0.479	0.530	0.325	0.317	0.332	0.360	0.340	0.380
11131	-1.116	-1.515	-0.716	0.206	0.165	0.248	0.429	0.398	0.459	0.377	0.369	0.384	0.408	0.388	0.428
12223	-1.027	-1.438	-0.616	0.210	0.172	0.249	0.396	0.367	0.425	0.339	0.332	0.347	0.369	0.349	0.389
23321	-2.077	-2.632	-1.522	0.136	0.094	0.178	0.388	0.357	0.418	0.248	0.241	0.255	0.275	0.256	0.295
21323	-1.585	-2.065	-1.105	0.151	0.111	0.192	0.380	0.350	0.410	0.265	0.258	0.273	0.293	0.273	0.312
32211	-1.689	-2.175	-1.203	0.139	0.098	0.179	0.372	0.342	0.402	0.256	0.249	0.263	0.282	0.263	0.302
21232	-2.145	-2.677	-1.613	0.061	0.020	0.103	0.328	0.296	0.360	0.299	0.292	0.307	0.325	0.305	0.345
22323	-2.128	-2.664	-1.593	0.046	0.005	0.086	0.297	0.265	0.330	0.207	0.200	0.214	0.228	0.209	0.248
22331	-2.230	-2.764	-1.697	-0.006	-0.047	0.035	0.270	0.235	0.304	0.220	0.213	0.227	0.241	0.221	0.260
33212	-2.669	-3.254	-2.084	-0.023	-0.063	0.018	0.258	0.224	0.292	0.183	0.176	0.189	0.202	0.183	0.221
11133	-2.760	-3.337	-2.182	-0.050	-0.091	-0.009	0.252	0.215	0.288	0.253	0.247	0.260	0.276	0.257	0.295
21133	-2.970	-3.584	-2.357	-0.067	-0.107	-0.026	0.222	0.187	0.258	0.224	0.217	0.230	0.244	0.225	0.263
23313	-3.203	-3.844	-2.562	-0.067	-0.107	-0.027	0.218	0.184	0.252	0.168	0.162	0.175	0.186	0.167	0.206
23232	-3.052	-3.647	-2.456	-0.092	-0.132	-0.052	0.208	0.172	0.244	0.184	0.178	0.191	0.204	0.184	0.223
33321	-3.200	-3.825	-2.576	-0.132	-0.170	-0.093	0.149	0.112	0.187	0.110	0.103	0.116	0.123	0.104	0.143
22233	-3.222	-3.833	-2.610	-0.145	-0.184	-0.106	0.149	0.110	0.187	0.157	0.150	0.164	0.172	0.153	0.192
32313	-3.307	-3.923	-2.690	-0.151	-0.190	-0.113	0.140	0.102	0.177	0.118	0.111	0.125	0.131	0.112	0.150
32223	-3.788	-4.458	-3.119	-0.185	-0.223	-0.146	0.113	0.074	0.152	0.128	0.121	0.135	0.142	0.123	0.162
32232	-4.084	-4.767	-3.401	-0.230	-0.269	-0.190	0.090	0.046	0.134	0.136	0.129	0.143	0.148	0.129	0.168
13332	-3.434	-4.035	-2.832	-0.222	-0.260	-0.184	0.075	0.033	0.118	0.138	0.132	0.145	0.152	0.132	0.171
32331	-4.812	-5.560	-4.063	-0.272	-0.310	-0.235	0.047	0.004	0.091	0.089	0.083	0.096	0.099	0.080	0.118
33232	-5.146	-5.922	-4.371	-0.327	-0.362	-0.292	-0.039	-0.085	0.007	0.049	0.042	0.056	0.055	0.035	0.075
33323	-5.914	-6.716	-5.112	-0.381	-0.415	-0.348	-0.096	-0.143	-0.049	0.024	0.017	0.031	0.028	0.008	0.049
33333	-8.883	-9.360	-8.407	-0.539	-0.554	-0.525	-0.304	-0.333	-0.275	-0.102	-0.107	-0.098	-0.108	-0.122	-0.095
Log Variance										-3.925	-3.943	-3.906	-3.819	-3.865	-3.773

## Discussion

In this paper, we introduce the episodic RUM and its coefficient estimator, which together provides a framework for health state valuation that is theoretically and econometrically consistent. The findings suggest a re-analysis of current health state valuation data and the potential merger of TTO and rank responses under a unified QALY estimator, specifically the exploded probit. To better understand this conclusion, we delineate the three major contributions of the episodic RUM.

The first contribution is the theoretical realization that under the conventional TTO approach, known as the instant RUM, the error scale in WTD and BTD responses is different by construction. As shown in equation 1, BTD error is divided by ten, and WTD error is divided by a number less than ten. Therefore, the instant RUM inflates the error of WTD responses, causing them to become more influential on the estimator and pulling the estimates down. Dolan's transformation of WTD responses (-*t*_2_/10) inadvertently causes the error scale to be equivalent, but the predictions lose internal consistency[12]. On the contrary, the episodic RUM assigns the same error scale, regardless of response type, and produces consistent results.

The second contribution is in convergent validity [11]. The episodic RUM predictions from the TTO responses strongly agree with predictions from the rank responses. In fact, this strength of agreement is larger than the agreement between rank predictions and instant RUM predictions with the Dolan transformation of WTD responses. The results confirm ranking and TTO to be closely related, suggesting the combination of both methods' strengths: the sound psychometric foundations and feasibility of ranking, and the face validity of TTO as it relates closely to the QALY paradigm. In a previous paper, Craig, Busschbach and Salomon show that rank predictions are essentially equivalent to VAS predictions (Lin's rho = 0.98); therefore, the results of this paper complementarily demonstrate convergent validity in the predictions for rank, VAS and TTO under the episodic RUM [5]. Furthermore, this evidence on the promise of the episodic RUM demonstrates that Dolan's arbitrary correction of negative responses is outmoded.

The third contribution is more practical. Under the assumption of normal errors, the episodic RUM implies an exploded probit estimator that integrates rank and TTO responses. This exploded probit estimator increases the power of valuation studies considerably by combining responses from two forms of discrete choice experiments: TTO and ranking. We demonstrate that the integration of rank and TTO responses is feasible and decreases the standard errors of the state value predictions. By merging a psychometrically strong instrument (i.e., ranks) with discrete choice data based on utility theory (i.e., TTO), predictions are more robust. However, we recognize the appeal of the nonparametric episodic RUM estimator (equation 5).

## Conclusion

The episodic RUM may replace the current paradigm in health state valuation, given that the instant RUM changes the error scale by response type; arbitrary corrections of WTD responses produce aberrant results; and the exploded probit allows the integration of TTO, rank, SG, and other discrete choice responses in a theoretically and econometrically consistent manner. In more practical terms, future valuation studies (e.g., EQ-5D five level version) may be statistically powered using a variety of discrete choice responses. The next step might be to re-estimate each country-specific valuation set using the episodic RUM and further examine duration effects in components and errors.

## Competing interests

The authors declare that they have no competing interests.

## Authors' contributions

BMC performed the statistical analysis. BMC and JJVB conceived of the study together, and each participated in its design. All authors read and approved the final manuscript.

## Author's information

BMC is an Assistant Member for the Health Outcomes & Behavior Program at Moffitt Cancer Center in Tampa, Florida and Courtesy Associate Professor in the Department of Economics at the University of South Florida, Tampa, Florida. BMC holds professional membership for the EuroQol Group, the International Health Economics Association, the International Society for Pharmacoeconomics & Outcomes Research, and the American Society of Health Economists.

JJVB is professor and vice director of the Department for Medical Psychology and Psychotherapy of the Erasmus MC in Rotterdam. JJVB holds professional membership for the EuroQol Group (chair of the foundation), the International Health Economics Association, and the International Society for Pharmacoeconomics & Outcomes Research

## References

[B1] Torrence GW, Kane Rl, Kane RA (1982). Multi-attribute utility theory as a method of measuring social preferences for health states in long-term care. Values and Long Term Care.

[B2] Patrick DL, Starks HE, Cain KC, Uhlmann RF, Pearlman RA (1994). Measuring preferences for health states worse than death. Med Decis Making.

[B3] Mullahy J (2008). The Anatomy of Healthcare Cost Distributions. 2nd Biennial Conference of the American Society of Health Economists.

[B4] Craig BM, Ramachandran S (2006). Relative risk of a shuffled deck: a generalizable logical consistency criterion for sample selection in health state valuation studies. Health Econ.

[B5] Craig BM, Busschbach JJV, Salomon JA (2008). Ranking, Time Trade-Off and Visual Analogue Scale Values for EQ-5D Health States. Under Review.

[B6] Busschbach JJV, Weijnen T, Nieuwenhuizen M, Oppe S, Badia X, Dolan P, Greiner W, Kind P, Krabbe P, Ohinmaa A, Brooks R, Rabin R, Charro Fd (2003). A comparison of EQ-5D time trade-off values obtained in Germany, The United Kingdom and Spain. The Measurement and Valuation of Health Status Using EQ-5D: A European Perspective.

[B7] Stalmeier PF, Busschbach JJ, Lamers LM, Krabbe PF (2005). The gap effect: discontinuities of preferences around dead. Health Econ.

[B8] Salomon JA (2003). Reconsidering the use of rankings in the valuation of health states: a model for estimating cardinal values from ordinal data. Popul Health Metr.

[B9] McCabe C, Brazier J, Gilks P, Tsuchiya A, Roberts J, O'Hagan A, Stevens K (2006). Using rank data to estimate health state utility models. J Health Econ.

[B10] Krabbe PF, Stalmeier PF, Lamers LM, Busschbach JJ (2006). Testing the interval-level measurement property of multi-item visual analogue scales. Qual Life Res.

[B11] Nunnally JC (1978). Psychometric Theory.

[B12] Dolan P (1997). Modeling valuations for EuroQol health states. Medical Care.

[B13] Gudex C (1994). Time Trade-Off User Manual: Props and Self-Completion Methods. Report of the Centre for Health Economics.

[B14] Kind P, Dolan P, Gudex C, Williams A (1998). Variations in population health status: results from a United Kingdom national questionnaire survey. Bmj.

[B15] Craig BM (2008). The duration effect: a link between TTO and VAS values. Health Econ.

[B16] Stalmeier PF, Lamers LM, Busschbach JJ, Krabbe PF (2007). On the assessment of preferences for health and duration: maximal endurable time and better than dead preferences. Med Care.

[B17] Goldberger AS (1964). Econometric Theory.

[B18] Drummond MF, Sculpher MJ, Torrance GW, O'Brien BJ, Stoddart GL (2005). Methods for the economic evaluation of health care programmes.

[B19] Mullahy J, Manning W, Sloan F (1995). Statistical issues in cost-effectiveness analyses. Valuing Health Care.

[B20] Shaw JW, Johnson JA, Coons SJ (2005). US valuation of the EQ-5D health states: development and testing of the D1 valuation model. Med Care.

[B21] Efron B (1977). The efficiency of Cox's likelihood function for censored data. Journal of the American Statistical Association.

